# Using Experimental Statistical Models for Predicting Strength and Deformability of Self-Compacting Concrete with Ground Blast-Furnace Slag

**DOI:** 10.3390/ma15124110

**Published:** 2022-06-09

**Authors:** Vadim Zhitkovsky, Leonid Dvorkin, Dmyrto Kochkarev, Yuri Ribakov

**Affiliations:** 1 Department of Constructing Products Technology and Material Science, National University of Water and Environmental Engineering, 33028 Rivne, Ukraine; v.v.zhitkovsky@nuwm.edu.ua (V.Z.); l.i.dvorkin@nuwm.edu.ua (L.D.); d.v.kochkarev@nuwm.edu.ua (D.K.); 2 Department of Civil Engineering, Ariel University, Ariel 40700, Israel

**Keywords:** self-compacting concrete, blast-furnace slag, superplasticizer, strength, modulus of elasticity, experimental-statistical model, concrete composition design

## Abstract

Ground blast-furnace slag is one of the waste products available in Ukraine and other countries. It is obtained at metallurgical enterprises in huge quantities and can be efficiently used for concrete production. The article is devoted to obtaining experimental-statistical models of the influence of technological factors that determine the composition of self-compacting concrete (SCC) based on ground blast-furnace slag and polycarboxylate superplasticizer on compressive strength, tensile strength, prismatic strength, elastic modulus and crack resistance. Analysis of the investigated factors’ influence on the specified SCC properties is carried out and positive influence of blast-furnace slag and superplasticizer simultaneous action on durability and deformation characteristics is studied. A design method of SCC composition design using the obtained mathematical models is developed. It allows for the consideration of a set of necessary parameters simultaneously. A numerical example is given.

## 1. Introduction

Applying effective plasticizers and superplasticizers in concrete compositions enable to obtain concrete mixtures with high workability (cast mixtures) and hardened concrete with high physical, mechanical and operational characteristics based on ordinary Portland cement and aggregates [1,2]. At the same time, along with obvious advantages, concrete mixtures with high workability have a number of disadvantages, such as loss of fluidity, delamination, slow hardening, etc. The effect of those disadvantages can be significantly reduced by using third-generation superplasticizers and highly dispersed silica-containing materials of man-made origin, especially silica fume [2,3].

Wide use of self-compacting concrete (SCC) in construction is quite attractive, but it is limited by the need to use highly active mineral admixtures and the lack of sufficient experimental basis for predicting its properties [3]. In turn, for proper structural design, data on deformability and crack resistance of such concrete are required, and the effect of its structural properties caused by high content of binders, active mineral admixtures and superplasticizers on structural behavior and durability should be investigated [4,5].

One of the widespread active mineral additives for concrete is ground domain granular slag [6]. Of all types of metallurgical slags used for producing construction materials, blast-furnace slag is the most widely used one. This is because blast-furnace slag has a chemical and mineralogical composition that is close to that of cement and has the ability to acquire hydraulic activity at rapid cooling [7].

Blast-furnace slag is formed in large quantities at metallurgical enterprises. It is an artificial silicate material formed during smelting metals from ore due to the high-temperature interaction of ore, fuel, floodplains and gaseous medium. For many years, a significant amount of blast-furnace slag has accumulated in dumps, occupied agricultural land and polluted the environment. Therefore, disposal of metallurgical slag is important for environmental protection and resource reuse worldwide.

SCC is usually characterized by high cement consumption [8], so the issue of efficient use of blast-furnace slag to replace part of cement, which is addressed in this study, is very important to improve the environmental friendliness of construction.

In order to use SCC in structural elements, the influence of technological factors on concrete deformability, crack resistance and behavior under loading is important. Development of proper methodology for predicting the SCC deformability at the concrete composition design stage is also relevant.

One of the most important and well-predictable concrete deformation characteristics is its modulus of elasticity. It is known [9] that ordinary and high-strength concrete modulus of elasticity closely correlates with its prismatic strength. Empirical dependences that interrelate those parameters have been proposed [10]. These dependences are included in various design codes. The empirical equations are often significantly different each from other by the equation form and also by coefficients, which vary in a rather wide range.

As known, due to the lower content of coarse aggregate, which creates a rigid frame, SCC has lower modulus-of-elasticity values [11]. For low-class concrete, the decrease in SCC modulus of elasticity is about 40% [12]. As the concrete class becomes higher, the difference between SCC modulus of elasticity and that of ordinary concrete decreases to 5%. It is obvious that various technological and structural factors cause significant changes in modulus of elasticity; therefore, it is difficult to find a close relationship, which could predict the SCC modulus of elasticity.

## 2. Research Aim, Scope and Novelty

In order to design composition of SCC with blast-furnace slag, taking into account a set of specified properties, it is important to obtain mathematical dependences allowing evaluation of technological and concrete composition factors’ impact on the required properties. The method of mathematical experiments planning [13,14] is effective to obtain such dependences. The method enables the receipt of regression equations by statistical processing of data obtained from the “active” experiment, i.e., the experiments are conducted according to a mathematically based scheme that helps to minimize the number of experimental points [15].

In order to identify the complex effect of active mineral additives and polycarboxylate-type superplasticizers on SCC strength and deformable properties in a wide range of strength classes, as well as to establish mathematical relationships between properties and technological factors, a planned experiment was conducted.

The novelty of this study is that based on the experimental results, mathematical models of strength and deformation characteristics of SCC based on blast-furnace slag are obtained. These models allow for a design of concrete composition that provides the required concrete characteristics at different hardening stages with minimum possible cement consumption.

## 3. Experimental Program

As variable factors were selected Portland cement consumption C, kg/m^3^ (X_1_), superplasticizer content SP, % (X_2_) and consumption of mineral additives (blast-furnace granulated slag) Sl, kg/m^3^ (X_3_). The concrete-mixture workability at the points of the experiment was maintained at the level of S5 (Slump—250–270 mm, which corresponds to the cone spread of 560–620 mm (F5 according to [16]). 204 cubic specimens of 100 mm × 100 mm × 100 mm and 48 prisms of 100 mm × 100 mm × 400 mm were prepared from the concrete mixture. Compressive strength (f_cm_, MPa), splitting tensile strength (f_ctm_, MPa) and modulus of elasticity (E_cm_ were determined at 1 and 28 days. Mechanical properties of concrete (strengths of cubes and prisms) under a single short-term load were determined by standard methods [17].

For determining the strength of concrete cubic specimens, the experiment was performed according to a three-level three-factor plan B_3_ [15] to obtain mathematical polynomial models of the form:

Y = *b*_0_ + *b*_1_X_1_ + *b*_2_X_2_ + *b*_3_X_3_ + *b*_11_X_1_^2^ + *b*_22_X_2_^2^ + *b*_33_X_3_^2^ + *b*_12_X_1_X_2_ + *b*_13_X_1_X_3_ + *b*_23_X_2_X_3_, (1)

where Y is the output parameter, X_1_…X_3_ are variables, *b*_0_…*b*_23_ are the equation coefficients.

Investigation of deformation characteristics was carried out in accordance with the two-level plan of the three-factor plan [15]. The type of mathematical model is:

Y = *b*_0_ + *b*_1_X_1_ + *b*_2_X_2_ + *b*_3_X_3_ + *b*_12_X_1_X_2_ + *b*_13_X_1_X_3_ + *b*_23_X_2_X_3_, (2)

The planning conditions are given in Table 1.

The following materials were used in the research: Portland cement CEM I 42.5 (Table 2), sand with M*_f_* = 2.0, locally available granite crushed-stone fraction 5–20 mm, blast-furnace granular slag, polycarboxylate-type superplasticizer PCE50 (LLC UA-Chemical, Ukraine). The experiment-planning matrix and the concrete composition at the experimental points are given in Table 3.

By statistical analysis of the experimental results (Table 3), mathematical models of the output parameters, as well as correlations between individual initial parameters were obtained. The coefficients of mathematical models are given in Table 4.

## 4. Results and Discussion

SCC with organic-mineral modifier, which includes polycarboxylate SP and blast-furnace granulated slag, had compressive strength in the range from 3.6 to 51 MPa after 1 day of hardening. As expected, factors X_1_ (cement consumption) and X_2_ (SP content), which cause a significant decrease in W/C, contribute to a significant increase in strength at 1 day.

**Figure 1:**
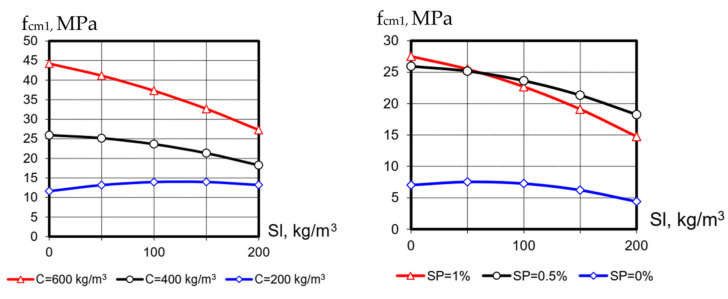
Dependence of compressive strength at 1 day on the blast-furnace-slag content.

The optimal content of the SP additive is significantly related to the cement consumption—at a minimum content of C just 0.5% of SP is enough to maximize the strength; at a maximum cement consumption the optimal SP content is 0.8–1.1. With the increase in the slag content, the optimal content of the additive and its effectiveness slightly decreases (Figure 1).

Increasing the blast-furnace-slag content (X_3_) mostly reduces the strength at 1 day of SCC hardening by 40–70%. As known [18], blast-furnace slag reacts rather slowly with cement-hydration products (primarily with portlandite), so a greater effect should be expected at longer hardening duration. At low cement consumptions, blast-furnace slag plays a positive role; there is an increase in f_cm1_ by 20–25%.

**Figure 2:**
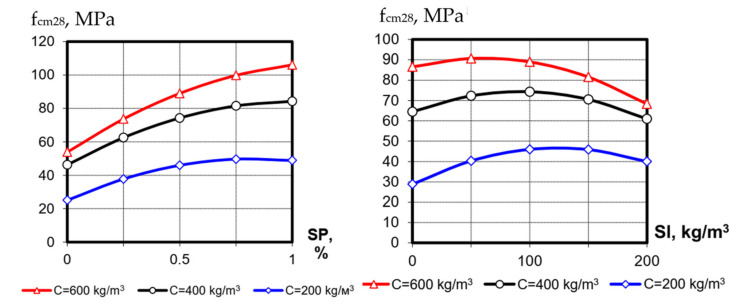
The influence of SP and blast-furnace-slag content at different levels of cement consumption on the SCC compression strength at 28 days (the value of the third factor in the graphs is at the average level (Table 1)).

The concrete classes by strength at 28 days that were investigated in the frame of the present research ranged from C8/10 to C 70/85. In general, the tendencies of the factors affect, detected for strength at 1 day, are similar to those at 28 days (Table 4, Figure 2). It should be mentioned that the efficiency of factor X_3_ (blast-furnace-slag consumption) becomes higher. The extreme influence of blast-furnace-slag content becomes more noticeable, which makes it possible to determine its optimal amount from the position of achieving maximum strength: at a cement consumption of 600 kg/m^3^ the optimal slag content from the strength viewpoint is 40–50 kg/m^3^ and it is 120–150 kg/m^3^ at cement consumption of 200 kg/m^3^.

**Figure 3:**
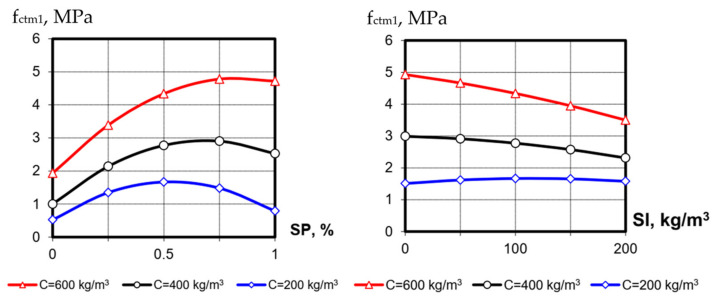
The influence of SP content and blast-furnace-slag content at different levels of cement consumption on SCC splitting tensile strength at 1 day (the value of the third factor on the graphs is at the average level (Table 1)).

**Figure 4:**
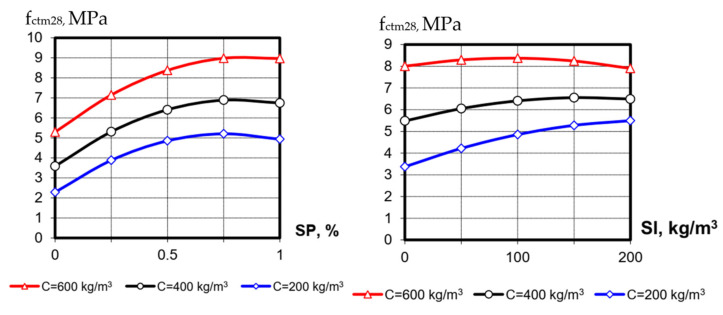
The influence of SP and blast-furnace-slag content at different levels of cement consumption on SCC splitting tensile strength at 28 days (the value of the third factor in the graphs is at the average level (Table 1)).

The values of splitting tensile strength at the 1st day (f_ctm1_) varied from 0.4 to 5.44 MPa and at 28 days (f_ctm28_), from 1.35 to 8.9 MPa. Following the mathematical models, the nature of the variable factors’ influence on splitting tensile strength is almost the same as for compressive one (Figure 3 and Figure 4). Only the more noticeable positive effect of blast-furnace-slag content on f_ctm_ at 28 days is noteworthy, especially at low cement consumption. As known [19], splitting tensile strength is mainly determined by the cement stone strength and its amount in concrete, so at low cement consumption, such an effect of increasing the total amount of binder with a long interaction of active additives and cement is expected.

**Figure 5:**
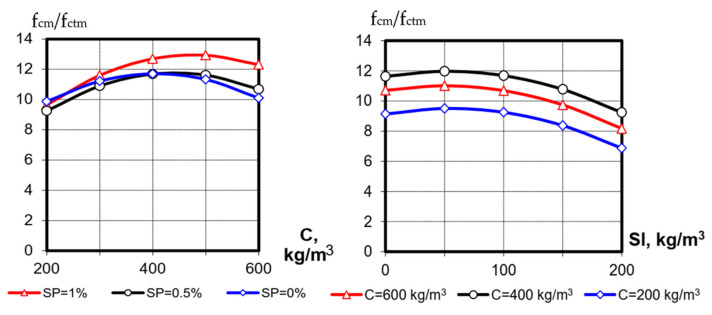
Influence of varied factors on the crack-resistance criterion f_cm_/f_ctm_.

One of the criteria for crack resistance of concrete is the ratio between compressive and tensile strengths f_cm_/f_ctm_. A decrease in this ratio indicates an increase in concrete resistance to cracking [9]. Using the calculated values from Table 3, a mathematical model of the ratio f_cm_/f_ctm_ at 28th days is obtained (Table 4). The main graphical dependences of the crack resistance index on the investigated factors are shown in Figure 5. The value of this criterion according to experimental data is in the range from 7.4 to 13.8. Analysis of the model shows that the achievement of the minimum criterion values is greatly facilitated by the increase in the blast-furnace-slag content (factor X_3_) at a minimum cement consumption (X_1_) (Figure 5). According to the available results [9], during hardening of cements with high slag content, a significant amount of low-basic calcium hydrosilicates is formed. As known [20], due to their nature these hydrosilicates are more deformable compared to highly basic calcium hydrosilicates, formed by hydration of Portland cement clinker.

Prismatic strength and modulus of deformation of SCC with organic-mineral modifier were studied by testing 100 mm × 100 mm × 400 mm prisms. The influence of the same three factors (cement consumption (X_1_) C, kg/m^3^, superplasticizer content (X_2_) SP, %, blast-furnace granular-slag consumption (X_3_) Sl, %) was studied according to the linear plan of the full factorial experiment [15]. The coefficients of the mathematical model are given in Table 4.

**Figure 6:**
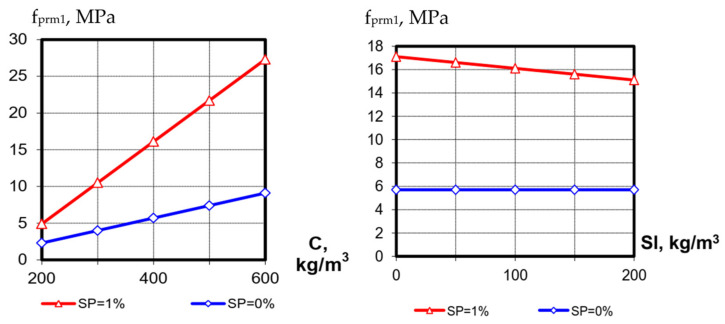
Influence of varied factors on SCC prismatic strength at 1 day (the value of the third factor on the graphs is at the average level (Table 1)).

The prismatic strength after one day of hardening ranged from 2 to 32 MPa, which corresponded to 78% of concrete cubes’ strength at the same age. The highest influence on the prismatic strength is caused by factors X_1_ (C) and X_2_ (SP), which first of all cause a decrease in W/C in transition from the lower to the upper level (Figure 6). Factor X_3_ (content of blast-furnace granulated slag) has little effect on the prismatic strength after one day of hardening.

**Figure 7:**
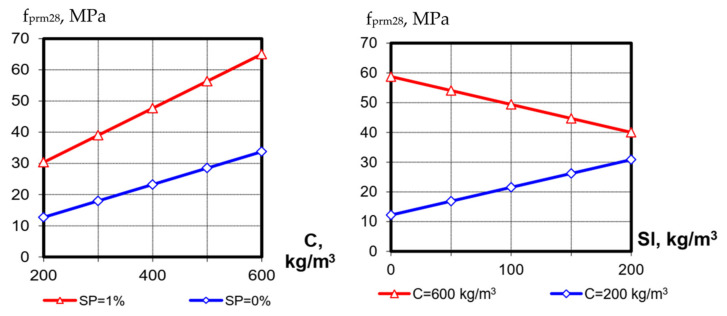
Influence of varied factors on SCC prismatic strength at 28 days (the value of the third factor on the graphs is at the average level (Table 1)).

After 28 days of hardening (Figure 7) interaction of factors X_3_ (slag content) with X_1_ (cement consumption) becomes more noticeable: for low cement consumption, an increase in slag content has a positive effect on concrete prismatic strength, while at high cement consumption the same increase in slag consumption causes a decrease in strength. As known [9], concrete deformations play a greater role in concrete prisms’ destruction (compared to the test of cubes), so the prismatic strength can be considered more as a concrete-deformation characteristic. The positive effect of the cement slag component at high values of W/C also confirms the structural role of low-basic calcium hydrosilicates, which are formed to a higher extent than in clinker cements.

**Figure 8:**
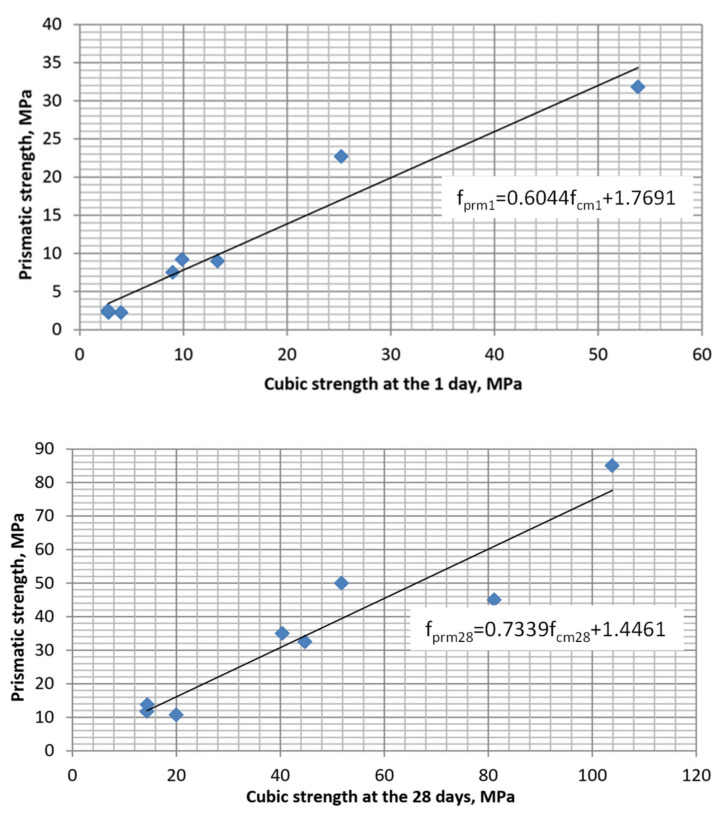
Correlation between the SCC cubic and prismatic strength.

The influence of other factors (cement consumption and SP content) on the prismatic strength at 28 days is similar to that after 1 day of hardening. The strength varies from 11 to 85 MPa. The mean value of the ratio between cubic and prismatic strength at 28 days is like after one day of hardening: 78%. The correlation between these concrete characteristics is shown in Figure 8.

The values of concrete modulus of elasticity at the first day were in the range from 10,000 to 27,000 MPa, at the 28th day—from 19,000 to 50,000 MPa. Analyzing the mathematical models, it should be noted that all three investigated factors significantly affect the SCC modulus of elasticity. The interactions between these factors are also significant.

**Figure 9:**
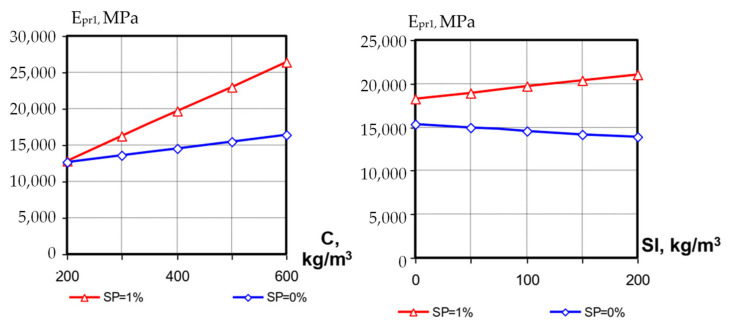
Influence of cement, slag and SP contents on SCC modulus of elasticity at 1 day.

**Figure 10:**
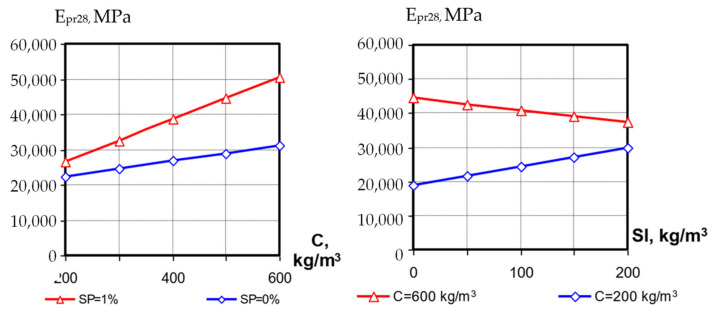
Influence of cement, slag and SP contents on SCC modulus of elasticity at 28 days.

At transition of factors X_1_ (C, kg/m^3^) and X_2_ (SP, %) from the lower level to the upper one the SCC modulus of elasticity increases on average from 10,000–12,000 to 20,000–26,000 MPa at 1 day and from 20,000–30,000 to 40,000–50,000 MPa at 28 days. The positive interaction coefficient of these factors (*b*_12_, Table 4) indicates a significant increase in the modulus of elasticity values with a simultaneous increase in cement consumption and superplasticizer content, both after 1 day and after 28 days of hardening. Factor X_3_ (slag consumption) on average has less effect on the modulus of elasticity than X_1_ and X_2_, but its effect is significantly enhanced by the simultaneous influence of other factors. At low cement consumption (C = 200 kg/m^3^) the increase in slag consumption increases E_pr_ by 1.5–1.7 times both at early hardening stages and at 28 days; however, at high cement consumption the same factor causes a significant reduction in SCC modulus of elasticity (Figure 9 and Figure 10).

The action of superplasticizer has a significant influence on the change of SCC modulus of elasticity vs. the blast-furnace-slag content; if in compositions without SP the positive effect of factor X_3_ is manifested only at low cement consumption, then the diluting and water-reducing action of polycarboxylate SP contributes to the positive effect of the slag component, and at higher cement costs it is probably leveling the significant increase in concrete-mixture water demand and causing formation of a denser concrete structure.

For predicting the concrete modulus of elasticity under loading at the age of τ days the most used dependencies have the following form:

(3) $$E_{c}=\frac{E_{m}R_{\tau }}{S+R_{\tau }},$$

where R_τ_ is compressive strength of concrete cubes after a certain curing duration (τ); E_m_ and S are empirical constants. Modern codes for structural design recommend the following values: E_m_ = 52,000; S = 23 [9].

According to the European Concrete Committee recommendation [9], the following dependence should be used:

(4) $$E=C{\left(R_{\tau }\right)}^{\gamma },$$

where C = 1900, γ = 0.5.

Different modifications of Equation (4) and coefficient values were proposed [10]. According to ACI-318-83 [21], concrete modulus of elasticity can be calculated as follows:

(5) $$E=4540\sqrt{f_{C}},$$

For high-strength concrete, the American Concrete Association has proposed a different equation [11]:

(6) $$E=3320\sqrt{f_{C}}+6900,$$

Deformability characteristics of concrete with modifying additives differ from the usual one. Elastic properties of modified concrete, including SCC, increase 1.4 times, and plastic ones decrease twice [10]. For modified concrete, the following dependence was obtained:

(7) $$E=5370.8\sqrt{f_{C}},$$

This dependence is fundamentally similar to Equations (3) and (4) and differs by a refined coefficient for modified high-strength concrete. The difference between the values of E calculated by Equations (3)–(7) increases (up to 35%) as the concrete strength increases [9].

**Figure 11:**
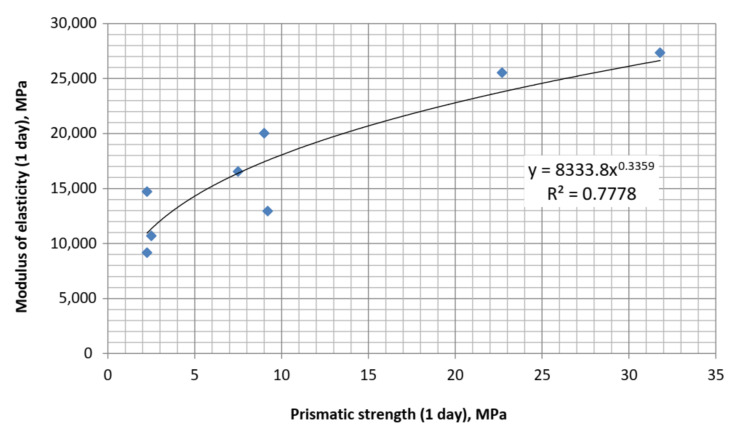
Correlation between the modulus of elasticity and prismatic strength at 1 day.

**Figure 12:**
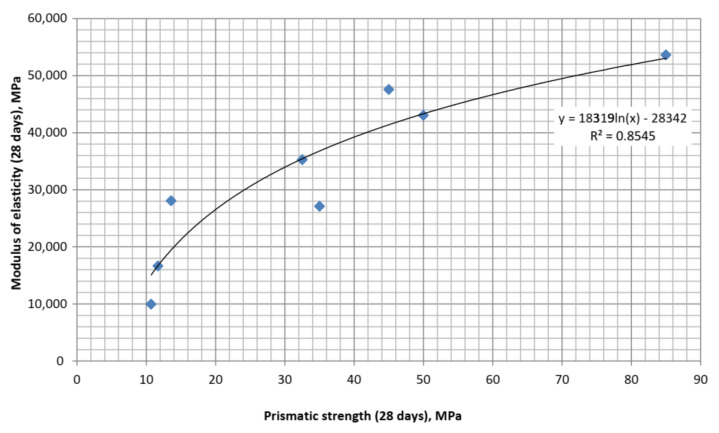
Correlation between the modulus of elasticity and prismatic strength at 28 days.

Based on the obtained data, the dependence between the modulus of elasticity of self-compacting concrete with a multifunctional modifier containing blast-furnace slag and superplasticizer was approximated (Figure 11 and Figure 12). The obtained equations have the following form:

- after 1 day of hardening:

(8) $$\mathrm{Epr}1=8334f_{\mathrm{pr}1}{}^{0.34},$$

- after 28 days of hardening:

(9) $$\mathrm{Epr}28=18319ln\left(f_{\mathrm{pr}28}\right)-28342$$

Table 5 shows the modulus of elasticity values, calculated by empirical Equations (1), (3)–(5) and (9) by strength indicators, as well as experimental values obtained for SCC. As it can be seen from Table 5, the average value of errors for calculations by all equations varies within a fairly narrow range: 18–35%. Significant differences in the errors obtained for calculations according to each of the equations (see Table 5 vertically) allows one to suggest that along with close correlation between strength and modulus of elasticity, which is noted by many authors [9,11,22,23], this indicator is significantly dependent on concrete qualitative composition, and especially the content of mineral and chemical additives that modify the cement stone structure. Comparing the data in Table 5, it becomes obvious that using correlations between strength and modulus of elasticity is appropriate only for approximate establishment of the required deformation characteristics, for example, for the design of reinforced concrete structures.

A method of creating and using multifactor dependences obtained, for example, by mathematical experiments planning has a higher resolution to perform tasks of predicting concrete properties at the composition design stage. The obtained experimental-statistical models with coefficients given in Table 4 enable us to predict a set of strength and deformation properties at certain values of the investigated factors: consumptions of cement, blast-furnace slag and superplasticizer. The use of mathematical optimization methods [16] and the corresponding software allows one to find the values of the above-mentioned factors that provide the necessary properties with minimal resource consumption [24].

**Numerical example**. Calculate composition of high-strength SCC (Slump—250–270 mm) using ground blast-furnace slag with a specific surface area of 270 m^2^/kg and polycarboxylate superplasticizer. The required indicators are that concrete class C40/50 (cubic strength at 28 days at least 64.3 MPa), cubic strength and modulus of elasticity at 1 day must meet the normative indicators for class C12/15: at least 19.3 MPa and 27,000 MPa, respectively.

Characteristics of raw materials: true densities of cement, slag, sand and crushed stone are 3100 kg/m^3^, 2900 kg/m^3^, 2650 kg/m^3^, 2700 kg/m^3^. The part of crushed stone in the SCC should not exceed 0.35.

## 5. Solution

1. 1. According to experimental statistical models of f_cm__1_, f_cm__28_, E_pr__1_ (see Table 4) using the methods of mathematical optimization [25], find the values of factors (consumptions of cement and slag, and superplasticizer content) that provide required indicators and satisfying the condition that the total cost of these components is minimal (Table 6). For calculations purposes, the following cost of components was assumed: cement—88 EUR/t, ground blast-furnace slag—27 EUR/t, superplasticizer—2.85 EUR/t. These cost values were taken on the basis of market prices in Ukraine on 10 April 2021.

**Table 6:** Optimization results for mathematical models.

Indicator	Units	Value
Optimal values of factors		
Cement consumption per 1 m^3^	kg	492
Slag consumption per 1 m^3^	kg	200
SP content	%	1
Concrete quality indicators		
Normative compression strength at 1 day	MPa	19.3
Calculated * compression strength at 1 day	MPa	21.2
Normative value of modulus of elasticity at 1 day	MPa	23,000
Calculated * value of modulus of elasticity at 1 day	MPa	23,000
Normative compression strength at 28 days	MPa	64.3
Calculated * compression strength at 28 days	MPa	81.0

* The values of indicators calculated according to mathematical models for corresponding optimal values of factors (cement, slag and SP contents).

2. According to the mathematical model of W/C (see Table 4), calculate the value of W/C at the optimal factors values and concrete-mix water demand:

W/C = 0.26, W = C·(W/C) = 492·0.26 = 126 *l*/m^3^

3. Find the crushed stone consumption from the condition that its volume should be 0.35 of concrete volume:

$$CS=0.35\cdot \rho_{CS}=0.35\cdot 2700=945\;\mathrm{kg}/m^{3}$$

4. Find the sand consumption:

$$S=\left(0.65-\frac{C}{\rho_{C}}-\frac{W}{\rho_{W}}-\frac{Sl}{\rho_{Sl}}\right)\cdot \rho_{CS}=\left(0.65-\frac{492}{3100}-\frac{126}{1000}-\frac{200}{2900}\right)\cdot 2650=786\mathrm{kg}/m^{3}$$

5. Find the superplasticizer content:

$$B_{SP}=\frac{C\cdot SP}{100}=\frac{492\cdot 1}{100}=4.92kg$$

Concrete composition per 1 m^3^ is:

Cement—492 kg; water—128 *l*; SP—4.92 kg; slag—200 kg; sand—780 kg; crushed stone—945 kg. A spreadsheet with the calculation performed can be viewed in Supplementary Materials (Table S1: Numerical example).

When using raw materials that differ from those used in the obtaining models (Table 4), it is effective to use the method of experimental-calculation adaptation [26], allowing to adjust the values of models’ coefficients considering laboratory control data of the required indicators.

## 6. Conclusions

The properties of self-compacting concrete with organic-mineral modifier, including blast-furnace granulated slag and polycarboxylate-type superplasticizer, have been studied. Mathematical models of cubic and prismatic strength, as well as modulus of elasticity at different concrete ages were obtained.

The combined action of blast-furnace slag and polycarboxylate superplasticizer contributes to an increase in the self-compacting concrete elasticity modulus by 1.5–1.7 times. A method for composition design of self-compacting concrete using blast-furnace slag and polycarboxylate superplasticizer is proposed. The method allows for the consideration of the strength of prisms and also the influence of technological factors that characterize the SCC composition when determining its modulus of elasticity.

The use of the obtained mathematical models enables the optimization of the SCC composition in order to provide the necessary strength and deformability characteristics at minimum cost of the final product.

## Figures and Tables

**Table 1:** Experiment-planning conditions.

No.	Factors		Variation Levels			Variation Interval
	Natural	Coded	−1	0	+1	
1	Cement consumption (C, kg/m^3^)	X_1_	200	400	600	200
2	Superplasticizer content (SP, %)	X_2_	0	0.5	0.1	0.5
3	Consumption of blast-furnace granulated slag (Sl, kg/m^3^)	X_3_	0	100	200	100

**Table 2:** Characteristics of Portland cement and blast-furnace granular slag.

Material	Chemical Composition, %								Specific Surface Area, m^2^/kg
	SiO_2_	Al_2_O_3_	Fe_2_O_3_	CaO	MgO	SO_3_	R_2_O *	L.O.I **	
Portland cement	21.80	5.12	4.11	65.80	0.95	0.63	0.85	0.81	340
Blast-furnace granular slag	39.52	6.49	0.12	47.13	3.10	1.74	-	-	270

Notes: * R_2_O = Na_2_O + K_2_O, ** L.O.I—loss on ignition.

**Table 3:** The results of determining the experiment output parameters.

No.	Coded Factors			W/C	Compressive Strength		Splitting Tensile Strength		Prismatic Strength		Modulus of Elasticity, MPa	
	X_1_	X_2_	X_3_									
					f_cm1_, MPa	f_cm28_, MPa	f_ctm1_, MPa	f_ctm28_, MPa	f_prm1_, MPa	f_prm28_, MPa	E_cm1_	E_cm28_
**1**	1	1	1	0.26	25.2	81.1	3.4	8.4	22.7	45.0	25,529	47,563
**2**	1	1	−1	0.25	53.8	103.9	5.4	8.9	31.8	85.0	27,335	53,612
**3**	1	−1	1	0.56	9.9	40.4	1.4	5.3	9.2	35.0	12,946	27,102
**4**	1	−1	−1	0.44	13.3	44.7	1.8	4.3	9	32.5	20,022	35,270
**5**	−1	1	1	0.63	9	51.8	1.1	6.65	7.5	50.0	16,545	43,033
**6**	−1	1	−1	0.73	4	20	0.4	2.3	2.25	10.7	9165	9947
**7**	−1	−1	1	1.08	2.8	14.3	0.4	1.95	2.25	11.7	14,709	16,633
**8**	−1	−1	−1	0.86	2.7	14.4	0.6	1.35	2.5	13.6	10,697	28,078
**9**	1	0	0	0.29	43.3	92.3	5.2	8.3	-	-	-	-
**10**	−1	0	0	0.56	10.3	46.7	1.3	5.35	-	-	-	-
**11**	0	1	0	0.29	23	90.8	2.7	6.6	-	-	-	-
**12**	0	−1	0	0.55	9.3	43.7	1.2	4.15	-	-	-	-
**13**	0	0	1	0.36	17.5	53.5	2.2	6	-	-	-	-
**14**	0	0	−1	0.30	29	76	3.6	6.4	-	-	-	-
**15**	0	0	0	0.31	22	71.3	2.5	6.1	-	-	-	-
**16**	0	0	0	0.31	22.4	70.9	2.5	6.3	-	-	-	-
**17**	0	0	0	0.31	21.8	70.5	2.4	6.0	-	-	-	-

**Table 4:** Coefficients of experimental-statistical models of output parameters.

	*b*_0_	*b*_1_	*b*_2_	*b*_3_	*b*_11_	*b*_22_	*b*_33_	*b*_12_	*b*_13_	*b*_23_
Experimental statistical models according to Equation (1)										
W/C	0.30	−0.21	−0.13	0.03	0.13	0.13	0.04	0.01	0	−0.05
f_cm__1_	23.6	11.67	7.71	−3.85	1.98	−8.67	−1.54	6.06	−4.63	−2.53
f_cm__7_	58.5	20.85	19.22	−2.12	−4.01	−10.96	−2.80	10.61	−5.89	1.04
f_cm__28_	74.3	21.51	19.00	−1.79	−6.81	−9.01	−11.56	7.11	−7.34	1.69
f_ctm__1_	2.8	1.34	0.76	−0.34	0.23	−1.01	−0.12	0.63	−0.38	−0.09
f_ctm__28_	6.4	1.76	1.58	0.51	0.21	−1.24	−0.42	0.26	−0.56	0.28
f_cm_/f_ctm_	11.68	0.72	0.49	−1.20	−1.71	0.51	−1.25	0.61	−0.07	0.40
Experimental statistical models according to Equation (2)										
f_prm__1_	10.9	7.3	5.2	−0.5	-	-	-	3.9	−0.5	−0.5
f_prm__28_	35.5	13.9	12.2	−0.03	-	-	-	3.5	−9.3	−0.144
E_pr1_	17,119	4340	2525	314	-	-	-	2449	−2534	1080
E_pr28_	32,655	8232	5884	928	-	-	-	3817	−4482	5831

**Table 5:** Comparison of SCC modulus of elasticity values calculated using different empirical dependences with experimental data.

No.	Experimental Data		Calculated Data, according to Equations E_pr28_, MPa Error (∆, %)				
	f_pr__28_, MPa	E_pr28_, MPa	(1)	(3)	(4)	(5)	(9)
1	45	47,563	30,455 36	53,182 12	29,171 39	36,030 24	41,392 13
2	85	53,612	41,857 22	100,455 87	37,509 30	49,518 8	53,043 1
3	35	27,102	26,859 1	41,364 53	26,541 2	31,775 17	36,788 36
4	32.5	35,270	25,882 27	38,409 9	25,827 27	30,619 13	35,431 0,5
5	50	43,033	32,103 25	59,091 37	30,376 29	37,979 12	43,322 1
6	11	9,947	15,057 51	13,000 31	17,911 80	17,814 79	15,585 57
7	12.3	16,633	15,890 4	14,477 13	18,520 11	18,799 13	17,557 6
8	13.8	28,078	16,835 40	16,250 42	19,211 32	19,916 29	19,673 30
Average error, %			26	35	31	24	18

## Supplementary Materials

The following supporting information can be downloaded at: https://www.mdpi.com/article/10.3390/ma15124110/s1, Table S1: Numerical example.
